# Chorioallantoic membrane tumor models highlight the effects of cisplatin compounds in oral carcinoma treatment

**DOI:** 10.1016/j.isci.2022.103980

**Published:** 2022-02-24

**Authors:** Patrizia Sarogni, Ana Katrina Mapanao, Alessandra Gonnelli, Maria Laura Ermini, Sabrina Marchetti, Claudia Kusmic, Fabiola Paiar, Valerio Voliani

**Affiliations:** 1 Center for Nanotechnology Innovation@NEST, Istituto Italiano di Tecnologia, Piazza San Silvestro 12, Pisa, Italy; 2 NEST-Scuola Normale Superiore, Piazza San Silvestro 12, Pisa, Italy; 3 Radiation Oncology Unit, Pisa University Hospital, Via Roma 67, Pisa, Italy; 4 Institute of Clinical Physiology, CNR, Via G. Moruzzi 1, Pisa, Italy

**Keywords:** Biological sciences, Molecular biology, Cancer

## Abstract

The European Society for Medical Oncology (ESMO) suggests the use of chemotherapy as neoadjuvant, adjuvant, and concomitant to surgery and radiotherapy for the treatment of oral carcinoma by depending on the cancer stage. The usual drug of choice belongs to the platinum compounds. In this context, the evaluation of the cancer behavior associated with the administration of standard or emerging cisplatin compounds supports the establishment of optimal cancer management. Here, we have assessed and compared the performance of cisplatin alone and contained in biodegradable nanocapsules on standardized chorioallantoic membrane (CAM) tumor models. The vascularized environment and optimized grafting procedure allowed the establishment of solid tumors. The treatments showed antitumor and anti-angiogenic activities together with deregulation of pivotal genes responsible of treatment resistance and tumor aggressiveness. This study further supports the significance of CAM tumor models in oncological research for the comprehension of the molecular mechanisms involved in tumor treatment response.

## Highlights

• Molecular evaluations support the establishment of optimal oral cancer management
• Alternative *in vivo* models agree to the European Parliament Directive 2010/63/EU
• These models are pivotal to recognize the mechanisms involved in treatment response

## Graphical Abstract

**Figure:**
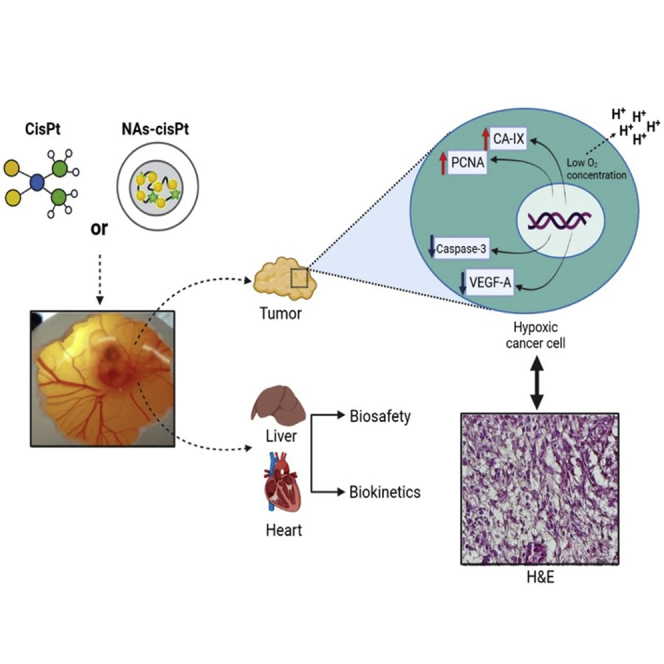

## Introduction

The epidemiology of head and neck squamous cell carcinoma (HNSCC) accounts for approximately 550,000 patients/y worldwide, resulting in more than 380,000 deaths/y (McDermott and Bowles, 2019). Although the excessive consumption of tobacco and alcohol are the main risk factors for the development of head and neck tumors with 5- to 25-fold increase in the onset of new cases, the incidence of human papilloma virus (HPV)-related cancer has shown a dramatic raise over the past 50 years (McDermott and Bowles, 2019). Advanced stages of HNSCCs are associated with increased tumor aggressiveness, which involve specific genetic and epigenetic landscapes such as loss of heterozygosity (LOH), gene deletions/deregulation, and DNA/RNA methylation (Robert et al., 2008; Romanowska et al., 2020). The genetic deregulation causes alterations in the major processes involved in the cell cycle, growth, motility, and migration. In particular, the aberration of some signaling cascades, among which the epidermal growth factor receptor (EGFR), Wnt/beta-catenin, transforming growth factor beta (TGF-β), PI3K-AKT-mTOR, Ras, and Stat, are associated with various HNSCC development mechanisms (Molinolo et al., 2009). In addition, the vascular endothelial growth factor (VEGF) significantly influences the angiogenesis and the growth/progression of HNSCCs (Mineta et al., 2000). Hence, the control of tumor angiogenesis has been an increasingly investigated therapeutic approach, and it is currently being evaluated for HNSCC (El-Kenawi and El-Remessy, 2013; Pozas et al., 2019; Tong et al., 2008; Vassilakopoulou et al., 2015). Actually, the treatment approach for HNSCCs depends on the stage of the disease and on the peculiar state of each patient (Matta and Ralhan, 2009). Chemotherapy is often employed in neoadjuvant, adjuvant, and metastatic setting with cisplatin as drug of choice also in combination with other drugs (Armand and Couteau, 1995). For instance, the first standard chemotherapeutic regimen introduced in the clinical practice (1970s) has been an administration of 100 mg/m^2^ cisplatin (day one) followed by an infusion of 100 mg/m^2^ of 5-fluorouracil (5-FU) for five consecutive days (Shabani and Larizadeh, 2015). Moreover, chemotherapy can be associated with radiotherapy in locally advanced tumor in order to increase the treatment efficacy and reduce the incidence of distal metastases (Rao, 2015). It is worth reporting that significantly longer survival rates have been observed in patients treated with multimodal chemoradiotherapy, potentially because of an enhanced locoregional control of the tumor (Shabani and Larizadeh, 2015; Sharma, 2020). Within this scenario, assessing the effects of monomodal chemotherapy on the neoplasms behavior can support the establishment of optimal cancer management.

Some emerging approaches may allow to further improve the efficiency of mono/multimodal chemotherapy. In this regard, noble metal nanotherapeutics have received special attention because of their unique behaviors (Vlamidis and Voliani, 2018). Beyond the peculiar interactions with radiations and potential combined actions, nanotherapeutics may exploit, for certain neoplasms, the disorganization of the tumor vasculature, which allow the passage and extravasation of nanoparticles within the tumor site by the enhanced permeability and retention effect (EPR) (Jain and Stylianopoulos, 2010). Despite the appealing features, noble metal nanomaterials with size above 6 nm lead to severe clearance issues caused by their accumulation within the primary excretory organs, such as the kidneys and bladder, or sequestration by Kupffer cells of the liver and macrophages in the spleen, thereby preventing their clinical translation (Cassano et al., 2018; Sun et al., 2014; Vlamidis and Voliani, 2018). In order to avoid the metal persistence, we have introduced a family of biodegradable nano-architectures (NAs) designed within the ultrasmall-in-nano approach (Cassano et al., 2015; Giannone et al., 2020). NAs are composed of polymeric aggregates of ultrasmall nanoparticles enclosed in approximately 100 nm silica nanocapsules (Cassano et al., 2017). Besides the elimination of the building blocks through the renal pathway, NAs can take advantage of both the modifiable silica surface to enable targeted delivery, and the functionalization of the inner cavity with moieties of interest (Avigo et al., 2017; Cassano et al., 2016, 2019a; Mapanao et al., 2018). Actually, NAs have been evaluated for the mono/multimodal chemo-photothermal treatment of HNSCCs (Cassano et al., 2019b; Mapanao et al., 2021b; Santi et al., 2020).

It is worth noting that the increasing availability and need for in-depth evaluation of innovative approaches for cancer detection and treatment have encouraged the establishment of reliable and advanced tumor models (Mapanao and Voliani, 2020). In this regard, tumor-grafted chorioallantoic membrane (CAM) models have emerged as alternative biological systems for oncological research (Mapanao et al., 2021a; Schneider-Stock and Ribatti, 2020). In fact, the highly vascularized membrane of this *in vivo* system is exploited to support the development of tumor xenografts and to explore the angiogenesis-related mechanisms and/or the anti-metastatic/antitumor activity of the treatment (Crespo and Casar, 2016; Deryugina and Quigley, 2008). Aside from an elaborated and ethical tumor representation, CAMs offer a variety of technical and practical advantages, including the rapid tumor mass formation, suitability to numerous cell lines, employment in non-aseptic laboratory, and cost-effectiveness (Mapanao et al., 2021a).

In the present study, the critical significance of CAM tumor models for chemotherapeutic evaluation is further demonstrated. Here, CAMs were grafted with SCC-25 cells, an HPV-negative HNSCCs line that usually results in particularly aggressive neoplasms. The tumor xenografts were employed to investigate and compare the effects of cisplatin alone and comprised as prodrug in NAs (NAs-cisPt). While previous studies focused on cell internalization, materials biodegradation, and cytotoxic effects of NAs-cisPt in 2D and 3D SCC-25 (Cassano et al., 2016; Santi et al., 2020), the present investigation evaluates its *in vivo* antitumor effects by assessing the tumor growth inhibition, tumor and organ histology, and the expression of cancer-related genes such as vascular endothelial growth factor-A (VEGF-A), proliferating cell nuclear antigen (PCNA), and caspase-3. Together with the potential angiogenesis-targeting effects of cisplatin and NAs-cisPt, the increased aggressiveness of surviving cancer cells is documented.

## Results

### Optimization of the *in vivo* model

In order to maximize the reproducibility and reliability of the *in vivo* model, different grafting procedures with a variable amount of cells (1 × 10^6^ cells/egg and 2 × 10^6^ cells/egg) and composition of the suspension (medium only, Matrigel only, and 1:1 Matrigel:medium mixture) were compared. While the conditions Matrigel alone and Matrigel-medium mixture generated solid visible tumors with different efficiencies, no tumor masses were observed on the CAMs grafted with cells suspended in medium alone, regardless of the cell density and for the whole duration of the study (Figure S1A). A considerably high tumor take (∼50%) until Embryo Day Development 17 (EDD17) was observed for the condition 2 × 10^6^ cells in 1:1 mixture, with respect to Matrigel alone (∼25.6%) and both conditions with 1 ×10^6^ cells (Matrigel alone: ∼41.7%; 1:1 mixture: 16.3%) (Figure S1B, *upper*). Unexpectedly, the inoculation of a higher number of cells did not result in larger tumors as observed in other neoplasms, such as the MNNG/HOS osteosarcoma cell line (Kunz et al., 2019).

The vitality of the embryos is another fundamental criterion to consider during tumor grafting because it affects the number of available samples for subsequent experiments. In addition, embryo survival can be used as an indicator of the toxicity during treatment evaluation. High embryo vitality was observed at EDD10 for all the conditions. On the other hand, only the samples grafted with 2 × 10^6^ cells in 1:1 mixture remained significantly viable until EDD17 (88.9%) with respect to 2 × 10^6^ cells in Matrigel only (Figure S1B, *bottom*).

Altogether, these data denote that the optimal grafting condition for the generation of SCC-25 models is 2 × 10^6^ cells in 1:1 Matrigel-medium mixture (Sarogni et al., 2021).

### Embryo vitality and tumor variations as predictors of treatment response

**Figure 1:**
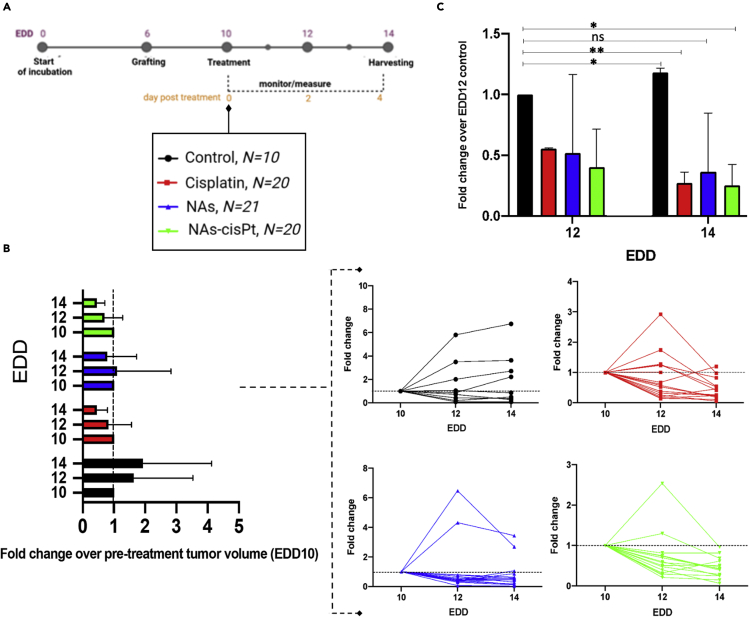
Evaluation of the treatments on tumor-bearing fertilized chicken eggs. (A) Scheme of the experimental set up. On EDD10 the eggs were randomized and distributed in four groups for topical administration of medium (control), cisplatin, NAs and NAs-cisPt. (B) Overall modifications of tumors size after the treatment (left) normalized on the mean of pre-treated tumor volumes at EDD10. Data are reported as mean ±SD of three independent experiments. No statistical differences were noted among the treatment conditions. Detail on each sample (right) for the four conditions during the experimental window. The dashed line refers to the fold change with respect to EDD10 and is equal to 1. (C) Volume fold change ratio for the four conditions. Data are reported as mean ±SD of three independent experiments. ∗p < 0.05 (Student’s *t* test). To see this figure in color, go online.

The optimized models were further employed to assess the performance of cisplatin alone and comprised in biodegradable nanocapsules as endogenously activable prodrug (NAs-cisPt). Head and neck cancer patients are usually managed with up to 100 mg/m^2^ of cisplatin (Osman et al., 2014). Thus, the clinically applied dosage of the drug has been employed in the *in ovo* treatments by considering the weight of the chicken embryo at EDD10 (2.3 g) and without applying the interspecies allometric scaling (Smith, 2019). Overall, each model has been treated with approximately 4 μg Pt (688 μM cisplatin in the application solution) (Nair and Jacob, 2016; Osman et al., 2014). As a control over the effect of the building blocks of the nano-architectures, another set of CAM tumor models was treated with standard NAs (*i.e.,* biodegradable nanocapsules without the prodrug) at the same amount of gold present in the administered NAs-cisPt (corresponded to ∼24 μg Au/egg). At EDD10, (*i.e.,* four days after grafting and just before the treatment) tumor-bearing CAMs were randomized into four conditions: Serum-free cell culture medium, cisplatin, NAs, and NAs-cisPt (Figure 1A).

Tumors treated with the serum-free medium were considered the control group (Figure S1C, *upper*). Tumor dimensions (in mm) were estimated from surface measurements, in which the length and width were defined as longer and shorter sizes, respectively. These values were utilized to calculate the tumor volumes by using a modified ellipsoid formula (Figure S1C, *bottom*) (Rovithi et al., 2017). However, this approach was not optimal to assess the effectiveness of the treatments because of the heterogeneity of the samples. Thus, the changes in tumor volumes were evaluated through the fold change value per each tumor.

Volume fold change refers to the ratio of the tumor volume at a certain post-treatment EDD (EDD 12 or 14) to the pre-treatment tumor volume (EDD 10). By this approach, a tumor retaining its volume will have a ratio equal to 1, a shrunken tumor <1, and an expanded tumor >1. Overall, medium-treated tumors demonstrated a slight but progressive increase of the volume fold change in EDD12 and 14 compared to EDD10, confirming that the application of medium alone did not affect the tumor growth. In contrast, tumors treated with cisplatin and NAs-cisPt revealed a progressive reduction of the volume fold change when compared to EDD10 while a small increase in volume fold change was recorded on EDD12 for the tumors treated with NAs, which subsequently decreased on EDD14 (Figure 1B, *left*). However, an individual analysis of each model highlighted that the overall size-reducing effect is time- and sample-dependent (Figure 1B, *right*). In order to better compare the volume fold change between the treatments, another normalization was applied. The average volume fold change values of tumors treated with cisplatin, NAs, and NAs-cisPt were compared with the average volume fold change of the medium-treated tumors on EDD 12 (Figure 1C). This approach intrinsically includes a normalization over the starting sizes of the tumors in all the conditions as well as the treatment effects. As expected, the ratios for the volume fold change were significantly reduced for cisplatin and NAs-cisPt conditions, while standard NAs did not significantly affect the size of the tumors. The evaluation of the treatments on the SCC-25-grafted CAM models was terminated at EDD14 to avoid further shrinkage of the tumor size at later time points, which can limit the availability of samples for the subsequent end-point assays (Figure S2A) (Kleibeuker et al., 2015; Sarogni et al., 2021). The volume of the harvested tumors was quantified with the same modified ellipsoid formula used for the surface measurements on CAMs. Overall, the average volumes of the harvested tumors were almost comparable to the *in ovo* tumor volumes calculated at EDD14 before the collection (Figure S2B). It is important to note that the accuracy of the tumor resection, which can be hardly distinguishable from the surrounding membrane and/or yolk, is crucial to avoid interferences from extra-tumoral tissues in subsequent end-point assays, such as the tumor weight analysis (Figure S2C).

### *Histological staining* analysis

**Figure 2:**
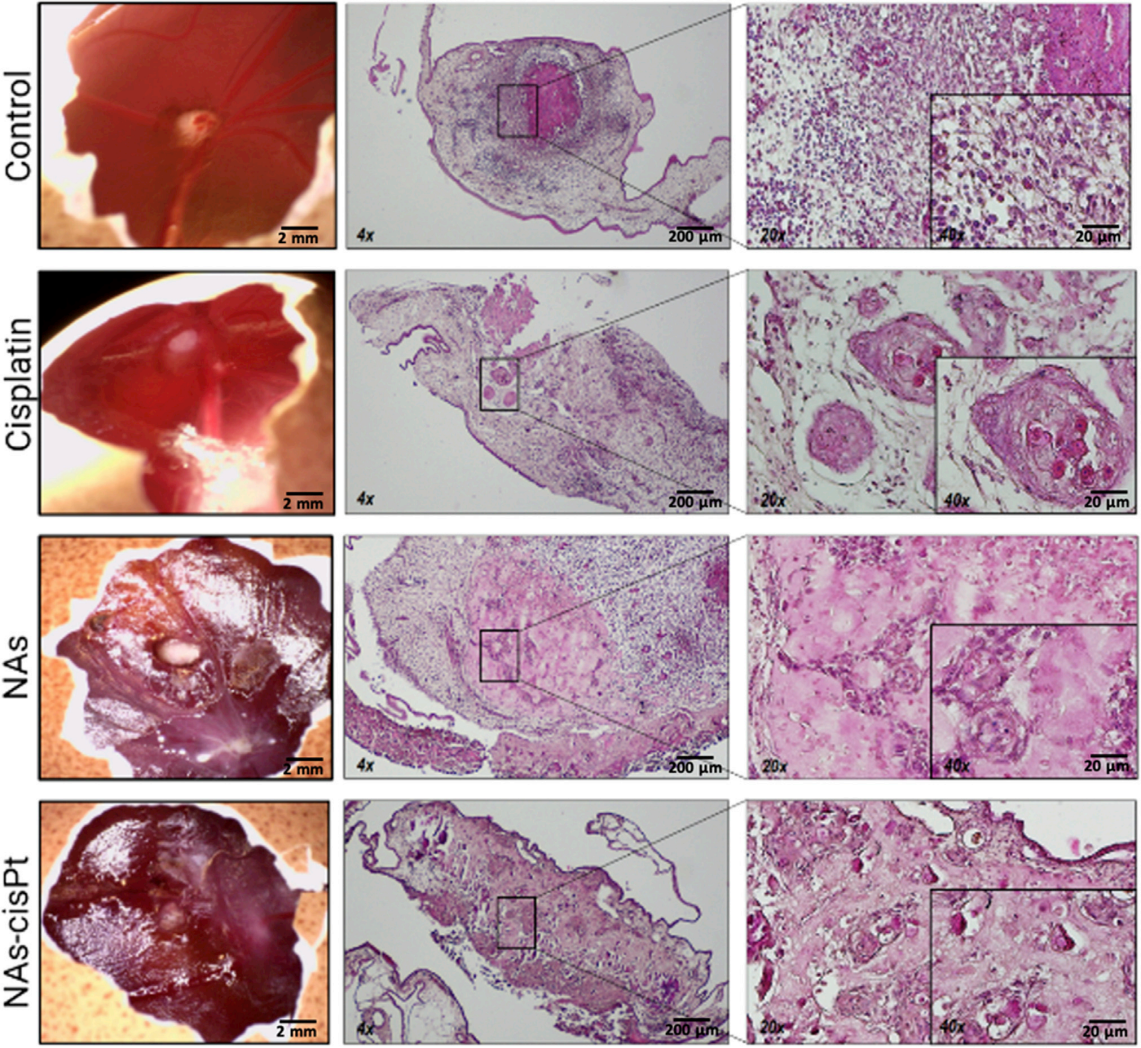
Tumor morphology after treatment. Representative images of tumor-bearing eggs (left column) for each condition at EDD14 (Scale bar: 2 mm). The associated histological images demonstrate the loss of tissue architecture and dispersed foci of cells regardless the type of treatment. (4× magnification, scale bar: 200 μm; 20× magnification, scale bar: 50 μm; 40× magnification, scale bar: 20 μm). To see this figure in color, go online.

The histological examination of tumor biopsies provides crucial information in the clinical practices (Chan, 2014). Therefore, the effects of the treatments on the morphology of the neoplasm growth in CAMs were evaluated through hematoxylin and eosin (H&E) staining. Although the medium-treated tumor did not show any obvious damages to the tissue structure and demonstrated a homogeneous population of cells in both morphology and distribution, all the treatments induced a chaotic and less compact tissue organization with dispersed and hardly identifiable cells (Figure 2). Noticeably, NAs-cisPt administration induced impairment throughout the whole section, whereas cisplatin and NAs-treated tumors contained undamaged areas (Figure S2D). These data suggest a potential synergistic effect of the gold and cisplatin comprised in NAs-cisPt, resulting in the complete disappearance of highly viable cancer cells.

### Hypoxia-induced chemoresistance promotes angiogenesis

**Figure 3:**
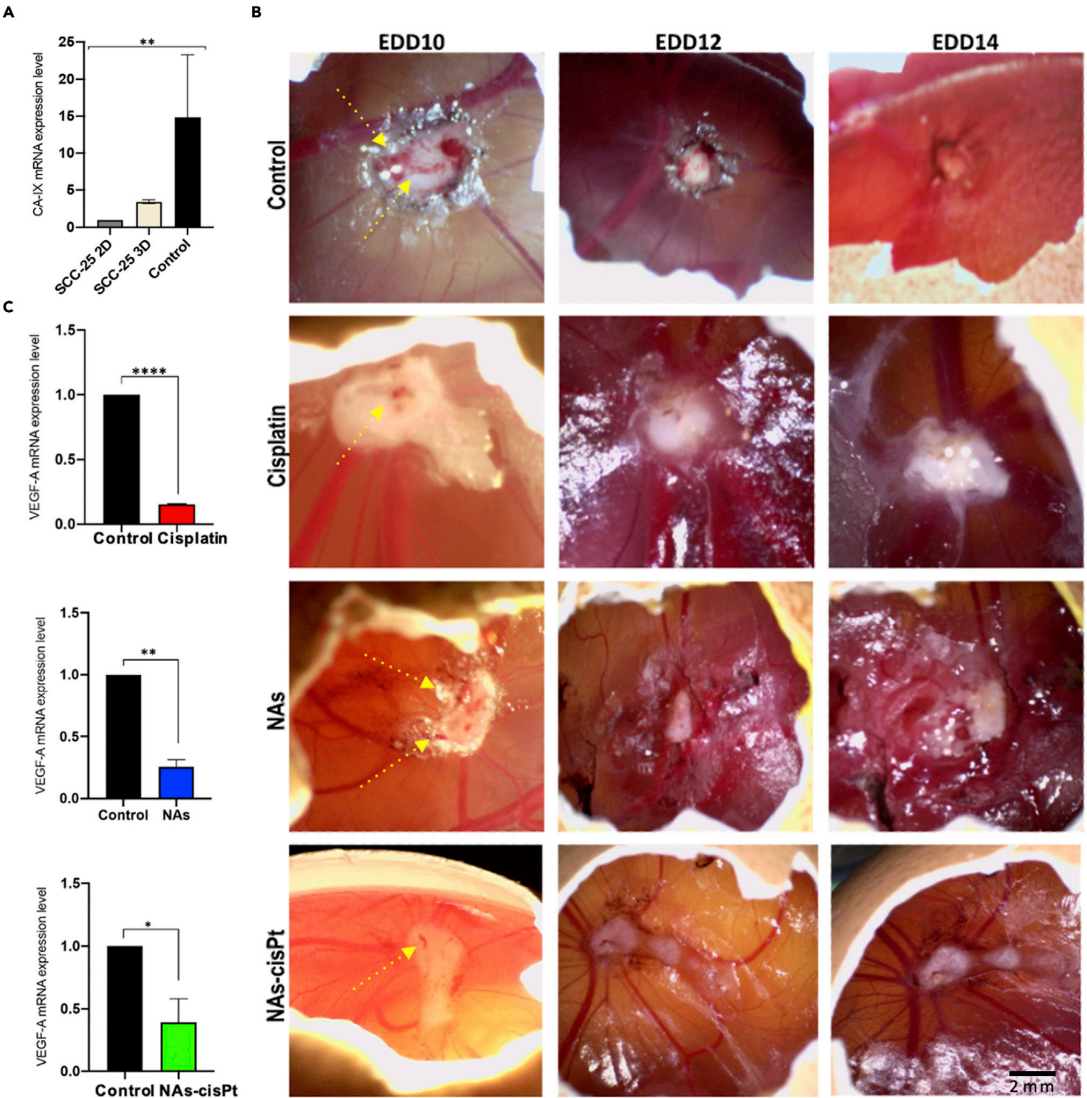
Effects of treatments on tumor vasculature. (A) Comparison of CA-IX mRNA expression levels between harvested CAM tumors and the related SCC-25 2D and 3D *in vitro* models. Data are reported as mean ±SD of two independent experiments. Significant differences were noted for each group. One-way ANOVA (Brown-Forsythe test, ∗p < 0.05). (B) Xenograft tumors demonstrate a highly vascularization at EDD10 (first column, yellow dashed lines). Blood vessels were undetectable after treatments, especially at EDD14; scale bar: 2 mm. (C) The expression level of VEGF-A was significantly reduced four days after the treatment. Data are reported as mean ±SD of two independent experiments. ∗p < 0.05 (Student’s t test). To see this figure in color, go online.

A low oxygen supply induces cancer cells to adapt their metabolism to the hypoxic environment, a non-physiological condition commonly observed in the majority of tumors (Muz et al., 2015). Therefore, the hypoxic feature of the tumors grown on CAMs was evaluated through the validation of the main hypoxia-responsive gene, *i.e.* carbonic anhydrase IX (CA IX) (Peridis et al., 2011). qPCR analysis revealed an elevated CA IX mRNA expression in tumor-grafted CAMs compared to the corresponding *in vitro* 2D and 3D models, supporting the suitability of CAM tumor model as an ideal *in vivo* system to mimic the characteristics of the original human solid tumors (Figure 3A) (Curry et al., 2014). The overexpression of CA IX is correlated with drug-resistance because of the reduced sensitivity of the cancer cells under hypoxic condition (Sowa et al., 2017). In addition, hypoxia was also found to trigger other tumor-promoting mechanisms, such as angiogenesis (Krock et al., 2011). Thus, the efficiencies of cisplatin, NAs, and NAs-cisPt in inhibiting hypoxia-mediated angiogenesis were evaluated. In this regard, microscopic observation of tumor vasculatures illustrated a progressive reduction of the blood vessels after the treatment except for the medium-treated tumors that exhibited abundant blood vessels up to EDD14 (Figure 3B). Next, the effects on angiogenesis modulation were confirmed through the analysis of VEGF-A, the major transcription factor involved in the formation of new blood vessels. In agreement with microscopic observation, the VEGF-A expression levels in the tumors exposed to the treatments were confirmed to be lower than the medium-treated tumors (Figure 3C).

### Hypoxia-induced chemoresistance promotes cancer cells repopulation in xenograft tumors

**Figure 4:**
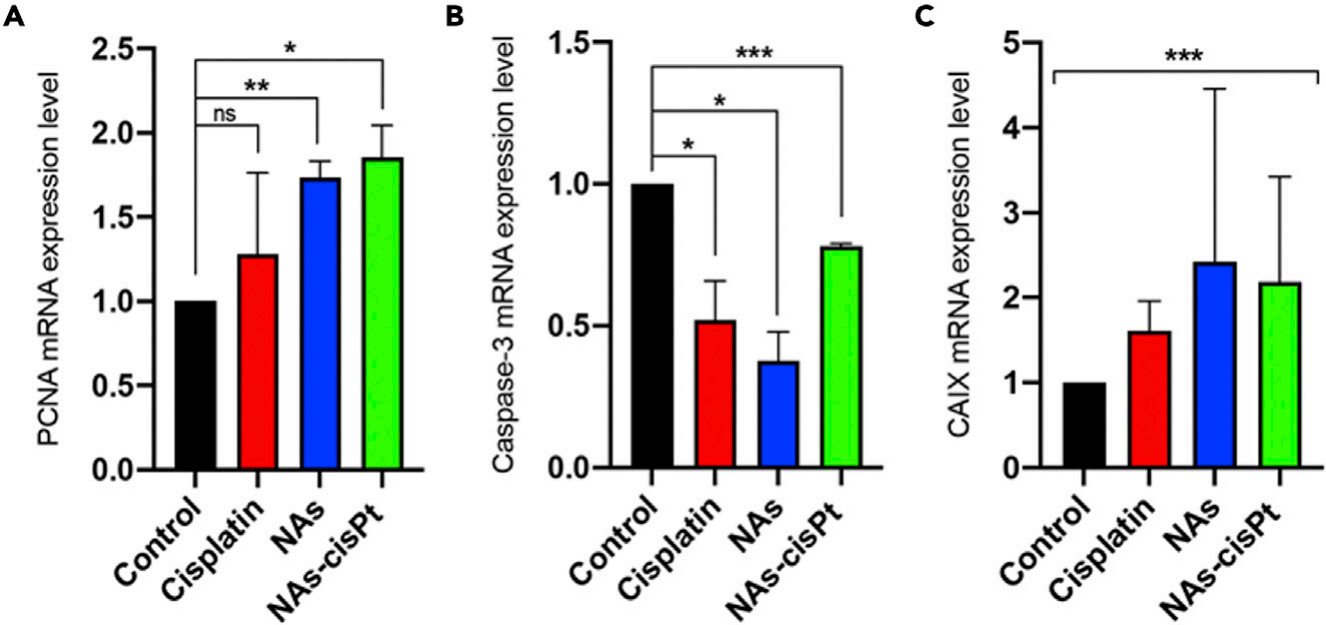
Gene’s expression analysis*.*Deregulation of PCNA (A) and caspase-3 (B) mRNA in CAMs was found for each treatment modality with respect to control group. Data are reported as mean ±SD of two independent experiments. ∗p < 0.05 (Student’s t test). (C) None of the treatment conditions restored the normal mRNA expression of CAIX. Data are reported as mean ±SD of two independent experiments. Significant differences were noted among the groups. One-way ANOVA (Brown-Forsythe test, ∗p < 0.05). *Gene expression studies were conducted on tumors collected four days after treatment (EDD14).* To see this figure in color, go online.

To determine whether the treatment modalities induce effects on cellular turnover, the genetic expressions of proliferating cellular nuclear antigen (PCNA), a marker of cell proliferation, and caspase-3, a protease involved in cellular apoptosis, were investigated. These genes are specifically used to assess the potential doubling time of viable cells of tumor samples harvested on EDD14. In general, albeit promising anti-angiogenic effects have been observed, alarming signs of tumor aggressiveness from the surviving fraction of cancer cells have also been identified. The mRNA expression level of PCNA gene was upregulated in treated tumors with respect to the tumors administered with the medium, together with decreased expressions of caspase-3 (Figure 4). Confounding dynamics were observed with the tumors treated with NAs-cisPt, which had the highest increase on PCNA expression but the least caspase-3 downregulation among the treatment conditions. In contrast, the increased PCNA expression caused by NAs was accompanied by a decreased caspase-3 expression (Figure 4). Notably, the increased proliferative index of tumor cells might be associated with hypoxia-induced chemoresistance, as each of the treatment modalities failed to reverse tumor hypoxia (Figure 4). Altogether, these data consistently demonstrated an increased cell repopulation, suggesting a compensating tumor-promoting response to the treatment.

### Biodistribution and biosafety evaluation

**Figure 5:**
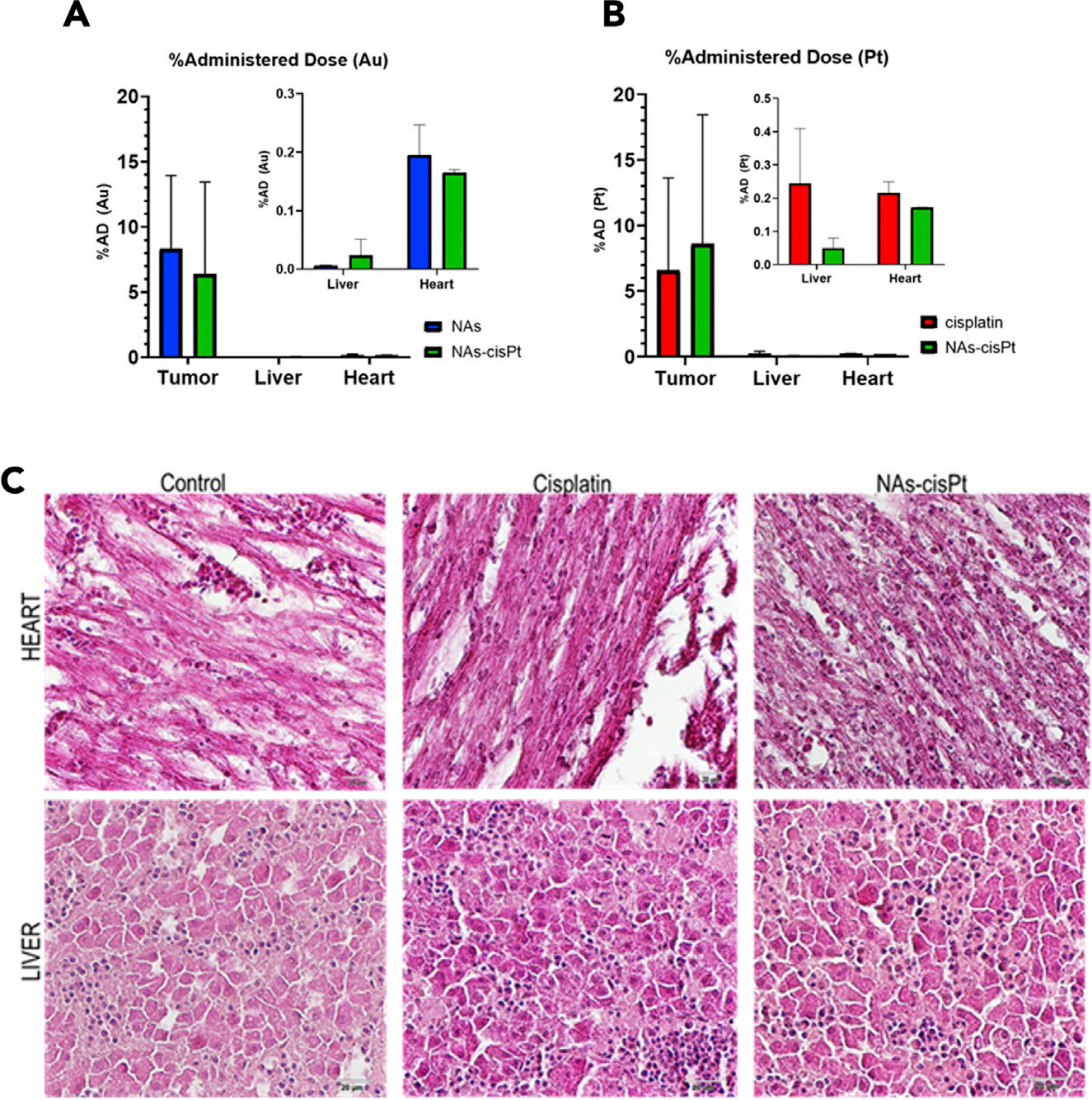
Evaluation of biocompatibility and biodistribution of nanomaterials in treated tumors and chick embryo’s organs. (A and B) Samples were processed for quantification of both gold (left) and platinum (right). The amounts of metals are reported as %Administered Dose (%AD). Detected amount of gold in: control = 0.042 ± 0.004 μg, free-cisplatin = 0.116 ± 0.054 μg. Detected amount of platinum in: control = 0.009 ± 0.003 μg, NAs = 0.019 ± 0.0026 μg. The data are reported as mean ±SD of the ICP-MS measurements, with at least two tissues per condition. Data were analyzed through two-way ANOVA, Šidák’|'s multiple comparisons test. p.value> 0.05. (C) Hematoxylin and eosin (H&E) staining of heart (*upper panel*) and liver (*bottom panel*) sections did not show any morphological alteration of the tissues or sites of inflammation. (Magnification 40×; scalebar 20 μm). To see this figure in color, go online.

CAM biological models were also employed to quantitatively assess the biodistribution and the biosafety profile of the treatments. The harvested tumors were processed for metal quantification of gold and platinum, which are present in the nano-architectures, through inductively coupled plasma-mass spectrometry (ICP-MS). Interestingly, the findings confirmed the comparable accumulation of gold in the tumors treated with NAs and NAs-cisPt, with %administration dose (%AD) values of 8.3% and 6.4%, respectively (Figure 5A). Meanwhile, the amount of platinum detected in the tumors treated with cisplatin and NAs-cisPt corresponded to 6.6% and 8.6% AD, respectively (Figure 5B).

To evaluate the biosafety profile of cisplatin and the nano-architectures, a qualitative histological analysis was performed on the hearts and livers of the treated embryos. The hearts were examined as cardiovascular complications are known to be among the adverse side effects of chemotherapy, whereas the liver was observed because of its role in cisplatin metabolism and the potential hepatotoxicity of drugs and nanomaterials (El-Sayyad et al., 2009; Isoda et al., 2013; Tamargo et al., 2015). No significant morphological alterations on cardiomyocytes and hepatocytes or inflammatory response were observed in both tissues (Figure 5C). This result is not surprising considering the negligible amounts of gold and platinum detected in the heart of treated embryos (Figures 5A and 5B; Table S1). Of interest, the non-persistent behaviors of the building blocks of the nano-architectures in liver was confirmed and in agreement with the literature (Cassano et al., 2019c; Mapanao et al., 2020). Moreover, free-cisplatin seems to be more easily accumulated in the liver with respect to the prodrug form included in NAs, highlighting the potential reduced toxicity associated with NAs-cisPt (Figure 5B).

## Discussion

The employment of *in vivo* models in oncological research is pivotal for both the identification of new therapeutic approaches and for the validation of the treatment efficacy. Xenograft tumor models represent a particularly appealing platform because of their ability to preserve and replicate the original tissue characteristics, such as the tumor microenvironment and vascularization, and their close correlation with patients (Pillai and Uthamanthil, 2017). In this regard, the employment of alternative *in vivo* models has been recently strongly suggested by the European Parliament Directive 2010/63/EU in order to strictly adhere to the 3Rs concept (Reduction, Refinement, Replacement) on the use of animals for scientific purposes (Törnqvist et al., 2014). In this context, we established a practical and ethical *in vivo* model based on the chicken chorioallantoic membrane (CAM) for advanced investigations into head and neck squamous cell carcinoma (HNSCC) (Sarogni et al., 2021).

In this study, the reliability and reproducibility of the tumor-grafted CAMs was confirmed using a specific HPV-negative cell line of HNSCC (SCC-25). It was further demonstrated that the amount of cells and the mixture composition used for the tumor implantation affect the grafting efficiency, with a significant impact on the vitality of the embryos (Sarogni et al., 2021). CAM tumor models also permit the evaluation of the anticancer efficacy of several therapeutic approaches, supporting their extensive use in oncological research to evaluate the behaviors of neoplasms and their response to treatments (Grace Intasa-ard and Birault, 2019). Here, cisplatin was used to validate the reliability of CAM as a preclinical *in vivo* model, thereby exploring whether the treatment response can be similar to clinical observations with HNSCC patients (Huysentruyt et al., 2010). Next, tumor-grafted CAMs were employed to evaluate the performance of an emerging nanotherapeutics, confirming the versatility of the chicken embryo model to assess different strategies for head and neck cancer treatment.

The effects of free-cisplatin, NAs and NAs-cisPt on the grafted tumors were first observed in terms of the average volume changes. Indeed, the volume variation of the primary tumor is one of the main prognostic factors, and determines the clinical treatment outcome of patients with locally advanced head and neck cancer (Romesser et al., 2014). It should be noted that we focused our investigation on monomodal chemotherapy in order to better evaluate its direct effects on the neoplasm behaviors. This approach would support the establishment of the optimal mono or multimodal management of HNSCC patients, especially at advanced stages (Hoebers et al., 2008; Surucu et al., 2016). The efficacy of cisplatin and NAs-cisPt, employed at an equivalent and clinically relevant dosage, were comparable. Meanwhile, the difference of platinum accumulation in the liver of the embryos treated with cisplatin and NAs-cisPt can be ascribed to the form of the therapeutics. Indeed, whereas free-cisplatin underwent the standard metabolization pathway, the prodrug comprised in NAs-cisPt is released with a double-endogenous control that improves the localized action of the drug (Santi et al., 2020). Interestingly, and in agreement with the literature, we also noted a slight antiproliferative effect in the samples treated with NAs that can be directly ascribed to the gold USNPs and that is not synergistic with cisplatin in NAs-cisPt treated samples (Arvizo et al., 2013).

Because of the highly vascularized biological model, the influence of the treatments on vascular (de)regulation have been assessed. Inhibition of tumor angiogenesis is an interesting strategy in cancer management, which relevance has been further denoted by the approval of VEGF-inhibitor bevacizumab for colorectal cancer (Hanahan and Weinberg, 2011). Aside from the non-invasive microscopic observation, which revealed a dynamic change in the global tumor vascularization, angiogenesis was also evaluated by monitoring the expression of the angiogenic biomarker VEGF-A. This gene is often upregulated in several types of cancer cells and induces proliferation, migration, and invasion of endothelial cells by promoting angiogenesis (Norrby, 2006). VEGF is highly expressed in HNSCCs and it is associated with lymph node metastasis and poor life expectancy, which suggest its potential as prognostic marker for this type of cancer (Chau and Haddad, 2016; Mineta et al., 2000). Noticeably, SCC-25 CAM tumor models consistently demonstrated the relevance of VEGF-A as an HNSCCs biomolecular marker, with its prominent expression in the control tumors. In contrast, VEGF-A was downregulated in all the treated tumors. This is in line with previous observations in which platinum-based drugs elicited anti-angiogenic activity in various mouse tumor xenograft models including renal, ovarian, lung, and gastric cancers (Lee and Wu, 2015; Li et al., 2014, 2016; Muscella et al., 2016). Meanwhile, the low VEGF-A expression in NAs-treated tumors was unexpected, yet previous studies have demonstrated the potential relevance of gold nanoparticles in anti-angiogenic treatment. In particular, Mukherjee et al. observed that gold nanoparticles (diameter ∼5 nm) caused angiogenic inhibition upon the interaction of the NPs with heparin-binding growth factors such as VEGF-165 and basic fibroblastic growth factor (bFGF). The effect was associated with the strong affinity of gold to thiols, which were mediated by the cysteine residues found in the heparin-binding domains of the growth factors (Mukherjee et al., 2005). The VEGF-limiting effect of NAs is an unanticipated result because previous studies have been performed solely on 2D and 3D cultured cancer cells and in the absence of tumor-supporting endothelial cells that have been proven to be vital in tumorigenesis. Nevertheless, no combinatorial effects of cisplatin and gold USNPs have been noted on VEGF-A downregulation in NAs-cisPt treated tumors. Hence, further investigations will be performed to clarify whether the VEGF-A downregulating effect of NAs-cisPt can be attributed to the gold USNPs or to the cisplatin prodrug, and to identify additional angiogenesis-relevant molecules, such as the epidermal growth factor (EGF). Although the treatments with cisplatin and NAs-cisPt have played a significant role on tumor volume reduction and angiogenesis inhibition, they also impaired the physiological cellular turnover. The combined increase of PCNA expression and downregulation of caspase-3 might be associated with an accelerated repopulation (AR) mechanisms upon treatments. This effect is supported by previous studies carried out on mouse models and patients, in which an increased repopulation during or after chemotherapy has been demonstrated (Kim and Tannock, 2005). It is worth pointing out that the low caspase-3 mRNA expression level may need further investigations to establish the presence of non-cleaved form of the protein, as the cleavage at the aspartate site is necessary to induce apoptosis (Pfeffer and Singh, 2018). However, tumor regrowth caused by the proliferation of the surviving cancer cells is also a common phenomenon in radiotherapy (Sowa et al., 2017). For example, the AR phenomenon is observed in the clinical practice with head and neck cancer patients undergoing radiotherapy (Withers et al., 1988). Indeed, the most recent study from randomized trials including 7,283 patients concluded that the rate of killing tumor cells modulate both the onset time and the rate of AR (Shuryak et al., 2018). Therefore, the portion of surviving cancer cells responds through AR when the tumor is exposed to a more intense dose fractionation regimen (Shuryak et al., 2018). A hypoxic environment is one of the main features of solid tumors and its association with drug resistance mechanism and cancer progression is extensively documented in many types of tumors (Muz et al., 2015; van Kuijk et al., 2016). Thus, CA IX was also investigated because of its role as a hypoxia-inducible gene. CA IX is also responsible for the pH balance between intracellular and extracellular compartment by favoring the migration and invasion of cancer cells (van Kuijk et al., 2016). Taken together, the aggressiveness of the surviving cancer cell population further denotes the requirement to concurrently address different cancer-promoting mechanisms. These findings confirm that mixed chemotherapeutics or multimodal approaches are essential for HNSCCs treatment and management. The correlation among hypoxia, CA IX overexpression, and the therapeutic response remains unclear. Nonetheless, these results exemplify the versatility of CAM tumor models for the evaluation of the therapeutic response at biomolecular level, and to identify potential mechanisms involved in treatment resistance.

### Conclusion

In summary, an optimized HPV-negative HNSCCs CAM model was employed to evaluate the anti-angiogenic and antitumor effects of emerging and standard cisplatin treatments. No combinatorial effects after NAs-cisPt application were recognized by a daily monitoring of neoplasms. Nonetheless, significant therapeutic effects were observed by histological analysis, in which the loss of structural arrangement was recognized. Furthermore, both cisplatin treatments elicit the action through VEGF-mediated mechanisms that resulted in tumor volume inhibition, but adverse drug resistance processes alter the cell turnover. Overall, tumor xenografts grown on CAM provide a promising alternative model for oncological research within the 3Rs principle in order to evaluate the performance of conventional/emergent treatments at a molecular level and promote the advancement of innovative approaches into the clinics.

### Limitations of the study

Our investigation demonstrates the critical importance of inserting alternative tumor models in the oncological research workflow. Despite tumor-grafted chorioallantoic membrane (CAM) models having emerged as one of the most interesting alternative biological systems for oncological research, some drawbacks need to be further optimized. For example, the lack of a human immune system does not actually allow to replicate the tumor-host immune system interactions. Noticeably, this drawback can be addressed by establishing CAMs from biopsies associated with autologous human leukocytes. Because of the short experimental window with CAMs, the follow-up period for the evaluation of the treatment effects might be too short. For this reason, performing the tumor grafting procedures at an early stage of development is crucial for these *in vivo* systems. The treatment is limited to a single dosage in the present work, although adequate for the assessment of various tumor-promoting mechanisms, such as angiogenesis, hypoxia, and proliferation.

## STAR★Methods

### Key resource table

**Table:** 

REAGENT or RESOURCE	SOURCE	IDENTIFIER
**Chemicals**		

Ammonia solution (32%)	Merck (EMPLURA®)	Cat#105426
cis-Diamineplatinum (II) dichloride (cisplatin)	Sigma-Aldrich	SKU 479306-1G
Ethanol (96.0–97.2%)	Sigma-Aldrich	SKU 24105-2.5L-M
Hydrochloric acid (concentrated; 30%)	Merck (SUPRAPUR®)	Cat#100318
Hydrogen tetrachloroaurate (III) trihydrate (ACS 99.99%)	Alfa Aesar	Stock # 36400
Nitric acid (concentrated; 65%)	Merck (SUPRAPUR®)	Cat#100441
Poly(L-lysine) hydrobromide (15–30 kDa)	Sigma-Aldrich	SKU P7890-1G
Poly(sodium 4-styrenesulfonate) (70 kDa, 30 wt. % in water)	Sigma-Aldrich	SKU 527483-100ML
Sodium borohydride	Sigma-Aldrich	Cat#452882
Tetraethyl orthosilicate	Sigma-Aldrich	SKU 131903-250 ML
Dehyol 95° (alcohol mixture)	Bio Optica	Cat#06-10070Q
Dehyol absolute (alcohol mixture)	Bio Optica	Cat#06-10077Q
Xylene	Bio Optica	Cat#06-1604F
Paraffin Bio Plast Plus	Bio Optica	Cat#08-7910
Mayer’s Hematoxylin	Bio Optica	Cat#05-06002E
Eosin Y 1% aqueous solution	Bio Optica	Cat#05-10002E
Permount Mounting Medium	Bio Optica	Cat#SP15500
DMEM/Ham’s F12 1:1 medium	Gibco	Cat#21041025
Fetal bovine serum (FBS)	Thermo Fisher Scientific	Cat#10500064
L-Glutamine	Thermo Fisher Scientific	Cat#A2916801
Hydrocortisone	Sigma-Aldrich	Ref H0888
Penicillin-streptomycin 100X	Thermo Fisher Scientific	Cat#15070063
Phosphate Buffered Saline (PBS)	Sigma-Aldrich	Ref D8537
Trypsin-EDTA (0.5%), phenol red	Thermo Fisher Scientific	Cat#25300054
Matrigel	Corning	Ref 354234

**Critical commercial assays**		

Nucleospin RNA plus Kit	MACHEREY-NAGEL	Ref 740984.50
iScript cDNA Synthesis Kit	Biorad	Ref 1708891
iTaq™ Universal SYBR® Green Supermix	Biorad	Ref 1725121

**Experimental model: Cell line**		

SCC-25 human cell line	ATCC	CRL-1628™

**Experimental model: Organism/Strain**		

Red Leghorn eggs	Local Poultry farming	Red Leghorn eggs

**Other**		

Egg incubator, 37.5°C/99.5°F, 60% humidity	FIEM	MG 140/200
Portable digital microscope	DinoLite	AM7915MZT
DinoCapture 2.0 Software	N/A	N/A
Nanodrop (UV5NANO)	Mettler-Toledo	N/A
Real-time PCR System	Applied Biosystems	7300
Inductively coupled plasma-mass spectrometer	Agilent	7700 series

### Resource availability

#### Lead contact

Further information and requests for resources and reagents should be directed to and will be fulfilled by the lead contact, Valerio Voliani (valerio.voliani@iit.it).

#### Materials availability statement

There are restrictions to the availability of nano-architectures because of the Material Transfer Agreement (MTA).

### Experimental model and subject details

#### Cell culture

Human squamous cell carcinoma SCC-25 was purchased from American Type Culture Collection (ATCC) and are from male origin. SCC-25 cells were cultured in a complete growth medium composed of a 1:1 mixture of Dulbecco’s Modified Eagle Medium and Ham’s F12 medium. The medium was supplemented with 10% fetal bovine serum (FBS), 4 mM L-glutamine, 1 mM sodium pyruvate, 100 U/mL penicillin, and 100 mg/mL streptomycin (Invitrogen), and with 400 ng/mL of hydrocortisone. The cells were maintained at 37°C in a humidified incubator with 5% CO_2_ atmosphere. SCC-25 cells were authenticated by short tandem repeat (STR) analysis and were free of mycoplasma contamination.

#### Tumor-grafted chorioallantoic membrane assay

Red Leghorn chicken eggs were incubated at 37°C in a fan-assisted humidified egg incubator (FIEM). A detailed step-by-step procedure has been reported elsewhere (Sarogni et al., 2021). Briefly, from Embryo Day Development 0 (EDD0) to EDD3 the eggs were positioned horizontally and automatically rotated to allow the natural formation of the air chamber. On EDD3, a small hole was made on the blunt end of each egg with fine tweezers and then the eggs were positioned vertically up to EDD6. At EDD6 a small window of about 1 cm^2^ was created and a total of 2 x 10^6^ SCC-25 cells diluted in a solution of cell culture medium plus Matrigel (Corning, Ref 354234) (ratio 1: 1) in a final volume of 25 uL were grafted on CAM. The treatment of tumor-bearing embryos was applied at EDD10. For cisplatin-treated tumors, a concentrated stock (1 mM) was made from *cis*-Pt(NH_3_)_2_Cl_2_ powder (Sigma-Aldrich 479306) first dissolved in 10 μL dimethyl sulfoxide and diluted with serum-free medium. The stock solution was then used to prepare the working solution with a final concentration of cisplatin equal to 688 μM. Meanwhile, an aliquot of NAs-cisPt was taken such that each tumor would also be treated with 688 μM cisplatin (corresponded to ∼4μgPt per egg). The amount of gold present in NAs-cisPt was first identified, and an aliquot of NAs was taken corresponding to the similar amount of gold (∼24 μg Au per egg). All materials were administered topically after resuspension in 30 μL serum-free medium. Embryo viability and tumor size were monitored every 2 days and tumor volume was calculated using a modified ellipsoid formula ½× (length × width^2^).(Rovithi et al., 2017) At EDD14, the chicken embryo movements were first slowed down by hypothermia (2 h at 4°C) and then the tumors were excised. After 24 h at 4°C, the organs were harvested. Samples were properly stored at −80°C for the following studies on mRNA expression or fixed in 4% paraformaldehyde for histological examination of the tissues. Samples for ICP-MS were stored at 4°C.

### Method details

#### Synthesis of the cisplatin prodrug-conjugated poly(L-Lysine)

The cisplatin prodrug c,t,c-[PtCl_2_(NH_3_)_2_(OH)(O_2_CCH_2_CH_2_CO_2_H)] was synthesized as in the following (Mapanao et al., 2021b; Santi et al., 2020). Cisplatin (0.40 g, 1.33 mmol) was suspended in milliQ water (10 mL) and H_2_O_2_ 30% (w/v) (14 mL, tenfold excess) was added. The mixture was stirred for 1h at 50 °C. Then it was cooled to 0 °C and saturated water solution of NaCl (10 mL) was added. The resulted pale yellow powder was collected by filtration and washed with cold water, ethanol and diethyl ether, and dried in a vacuum pump, yielding c,t,c-[PtCl_2_(OH)_2_(NH_3_)_2_] (223 mg, 0.67 mmol, 50%). To a solution of c,t,c-[PtCl_2_(OH)_2_(NH_3_)_2_] (0.2 g, 0.6 mmol) in anhydrous dimethyl sulfoxide (DMSO, 16 mL) was added succinic anhydride (0.06 g, 0.6 mmol) and the reaction mixture was stirred at room temperature for 12 h. The solution was freeze-dried and acetone (10 mL) was added to precipitate a light yellow solid, which was collected by filtration and washed several times with acetone, diethyl ether, and then dried in a vacuum pump, yielding c,t,c-[PtCl_2_(NH_3_)_2_(OH)(O_2_CCH_2_CH_2_CO_2_H)] (0.16 g, 0.37 mmol, 62%). The prodrug was covalently linked to poly(L-lysine) (PL) as in the following. 12 mg of the prodrug were dissolved in phosphate-buffered saline (PBS 1X, pH 7.4, 100 μL) and mix for 20 min with a freshly prepared solution of 1-Ethyl-3-(3-dimethylaminopropyl)carbodiimide (EDC) and N-Hydroxysuccinimide (NHS), also prepared in PBS (100 μL) (25 mg EDC/15 mg NHS). Then, the mixture was added with 750 μL of PL aqueous solution (15–30 kDa; equivalent to 30mgPL). The resulting mixture was incubated overnight while shaking (700 rpm) at room temperature. Then, the cisplatin prodrug-functionalized PL product (PL-cisPt) was recovered through filtration using Amicon 10 kDa centrifugal filter. The product was washed thrice with milliQ water and was finally resuspended in 1 mL milliQ water. The product was stored at −20°C until needed.

#### Synthesis of gold ultrasmall nanoparticles and polymeric arrays

Gold ultrasmall nanoparticles (USNPs) with an average diameter of 3 nm were synthesized by adding 200 μL of aqueous solution of tetrachloroauric (III) acid (HAuCl_4_; Alfa Aesar, ACS 99.99% metal basis; stock: 10 mg/mL) and 10 μL poly(sodium 4-styrene sulfonate) (PSS; 70 kDa; 30% aqueous solution) to 20 mL of milliQ water. Then, 200 μL of sodium borohydride (stock: 8 mg/mL) was quickly added while the solution with gold salts was vigorously stirring. The solution was allowed to stir vigorously for another 2 min and was further aged for 10 min. Following this, 165 μL of PL-cisPt (for NAs-cisPt) or 75 μL of PL stock solution (15–30 kDa; 40 mg/mL stock; for standard NAs) was added to the solution containing the gold USNPs, and the solution was incubated for 20 min. The Au USNP polymeric arrays were finally collected by centrifugation at 16873 × g for 3 min, then resuspended in milliQ water (2 mL).

#### Synthesis of nano-architectures

A modified Stöber process was followed to grow a silica nanoshell on the periphery of Au polymeric arrays. Ethanol (70 mL) and ammonia solution (2.4 mL; Merck, 32%) were first mixed, and added with 40 μL of tetraethyl orthosilicate just before 2 mL of the Au USNP polymeric arrays were also added. The solution was incubated for 3 h at room temperature, under moderate shaking. The resulting nano-architectures (NAs-cisPt or standard NAs) were collected through centrifugation at 3220 × g for 30 min. After discarding the supernatant, the products were resuspended in ethanol, sonicated, and spun at 16873 × g for 3 min. The supernatant was then discarded, and another series of washing was performed. Then, larger NAs were removed through a short cycle of centrifugation (15 s or until rotational speed reached 16873 × g). The supernatant was saved and again spun at 16873 × g for 3 min to collect the final products (standard NAs or NAs-cisPt), which was stored in 1 mL ethanol.

#### Inductively coupled plasma-mass spectrometry (ICP-MS) measurements

From a known stock volume of standard NAs or NAs-cisPt, an aliquot was taken and placed in a 10-mL borosilicate glass vessel. The sample was digested using freshly prepared aqua regia (3:1 molar ratio of ICP-MS analysis grade concentrated hydrochloric acid and nitric acid). The sealed vessels were placed in CEM Discover SP-D for further digestion under microwave irradiation (200°C/15 min). The digested NAs samples were diluted with 3% nitric acid solution, and the volume was adjusted to 5 mL. For biological samples, the harvested tissues were first dried overnight at 80°C. After cooling down to room temperature, the dried samples were weighed, transferred to 10-mL pressure vessels, and digested in nitric acid (∼3 mL) at 150°C for 30 min. The acid was evaporated, and the samples were again digested, this time using freshly prepared aqua regia. Finally, the digested tissue samples were dried and resuspended to a final volume of 3 mL with 3% nitric acid solution. The amounts of metals (gold and platinum) were determined through ICP-MS Agilent 7700, using standard calibration curves.

#### Quantitative real-time PCR

Total RNA was extracted from the harvested tumors using Nucleospin RNA plus Kit (740984.50 MACHEREY-NAGEL) following the manufacturer’s instructions. The extracted RNA was used immediately or stored at -80°C, and was quantified using a nanodrop instrument (UV5NANO Mettler-Toledo). Five hundred nanograms of RNA were reverse transcribed for cDNA synthesis with iScript cDNA Synthesis Kit (1708891 BIORAD). Reverse transcription of the RNAs was followed by quantitative real-time PCR (Q-PCR) performed with iTaq™ Universal SYBR® Green Supermix (1725121 BIORAD). Thus, 500ng of cDNA was diluted 1:10 to have a final concentration of 2.5 ng/μL and 1–2 μL of the dilution was used for the amplification of each gene. The reactions were visualized by SYBR Green Analysis on Applied Biosystem Instrument (7300). Primers used for gene analysis were the following: CAIX-Forward: 5′-CCTCAAGAACCCCAGAATAATGC-3′, Reverse: 5′-CCTCCATAGCGCCAATGACT-3′; PCNA-Forward: 5′-CGGATACCTTGGCGCTAGTA-3′, Reverse:5′-CACTCCGTCTTTTGCACAGG-3′; Caspase-3-Forward: 5′-CAAACTTTTTCAGAGGGGATCG-3′, Reverse: 5′-GCATACTGTTTCAGCATGGCAC-3′; VEGF-A-Forward: 5′-GGGCAGAATCATCACGAAGT-3′, Reverse: 5′-TGGTGATGTTGGACTCCTCA-3′; GAPDH-Forward: 5′-AGAAGGCTGGGGCTCATTT-3′, Reverse: 5′-AGTCTTCTGGGTGGCAGTGAT-3′. All samples were assayed in duplicate. The recommended thermal cycling for the amplification is as follows: 95°C for 10 min, 40 cycles at 95°C 15 s, 60°C for 30 s, 72°C for 30 s. To calculate the relative expression level, the 2^-ΔΔCT^ method has been used (Livak and Schmittgen, 2001).

#### Histological examination and imaging

Tumors fixed in paraformaldehyde for 24 to 48 h were rinsed in running tap water for 10 to 15 min. Then, samples were dehydrated through a series of washings in increasing alcohol concentration, followed by three changes of 100% alcohol. Tissues were cleared in xylene for 12 min, and then immersed in paraffin wax for 10 min, followed by other two paraffin changes for 5 min each. The samples were then embedded in paraffin blocks. Serial sections with 5–6 μm thickness were cut using a rotary microtome, placed on slides, and heated overnight at 40°C under forced ventilation histology oven. Then, the sections were cleared from paraffin after two changes in xylene, 6 min each. The tissue was rehydrated through another series of washing, this time with decreasing alcohol concentration. The re-hydration step was finalized with a rinse in distilled water for at least 5 min. The slides were stained with Mayer’s hematoxylin solution for 5 min, followed by a 10-min rinsing step in running tap water. Then, the same slides were stained with eosin Y aqueous solution for 2 min and then rinsed with distilled water. The slides were then heated at 40°C for 40 min and dipped twice in xylene. A Permount mounting medium was dropped and the coverslip was added. Histological images were acquired using a light microscope (Olympus BX43, Japan) and digitized using an RGB video camera (Olympus DP 20, Japan).

### Quantification and statistical analysis

#### *In vivo* studies

All error bars in graph for Figure S1B represent data reported as mean ± standard deviation of 2 independent experiments, with at least 4 eggs per condition. The significance of differences among the experimental groups (*e.g*., different grafting procedures) was assessed with two-way ANOVA, Tukey’s multiple comparison test, ∗p < 0.05. In Figure 1C, the significance of variation between the control group (EDD10) and each treated group (EDD12) was assessed with unpaired Student’s t-test in which data are reported as mean ± standard deviation of 3 independent experiments, ∗p < 0.05.

Biomolecular comparison of CAIX gene expression in Figure 3A was evaluated with one-way ANOVA; the Brown-Forsythe test was applied, ∗p < 0.05, and data are reported as mean ± standard deviation of 2 independent experiments. The significant deregulation of VEGF-A, PCNA and Caspase-3 expression level in Figures 3C, 4A and 4B was assessed with unpaired Student’s t-test and data are reported as mean ± standard deviation of 2 independent experiments, ∗p < 0.05. In Figure 4C the CAIX was re-evaluated after treatments and none of them restored its normal mRNA expression level. Significant differences among groups were assessed with one-way ANOVA; the Brown-Forsythe test was applied, ∗p < 0.05.

The data of nanomaterials biodistribution, a criterion for evaluating their toxicity, are reported as mean ± standard deviation of the ICP-MS measurements, with at least 2 tissues per condition, p.value>0.05. The significance of variation was determined through two-way ANOVA and Šidák’s multiple comparisons test was applied, p.value>0.05.

## Data Availability

• All data reported in this paper will be shared by the lead contact upon request • This data does not report original code • Any additional information required to reanalyze the data reported in this paper is available from the lead contact upon request
