# Supplementary material for: Chorioallantoic membrane tumor models highlight the effects of cisplatin compounds in oral carcinoma treatment
Source: iScience. 2022 Feb 24;25(3):103980. doi: 10.1016/j.isci.2022.103980 (PMC8924639; doi:10.1016/j.isci.2022.103980)
Supplement: Document S1. Figures S1 and S2 and Table S1 [file mmc1.pdf]

iScience, Volume *25*

Supplemental information

Chorioallantoic membrane tumor models highlight the effects of cisplatin compounds in oral carcinoma treatment

Patrizia Sarogni, Ana Katrina Mapanao, Alessandra Gonnelli, Maria Laura Ermini, Sabrina Marchetti, Claudia Kusmic, Fabiola Paiar, and Valerio Voliani

Figure S1. Evaluation of the optimization conditions on tumor take rate and embryo vitality and effects of the treatments, related to Figure 1.

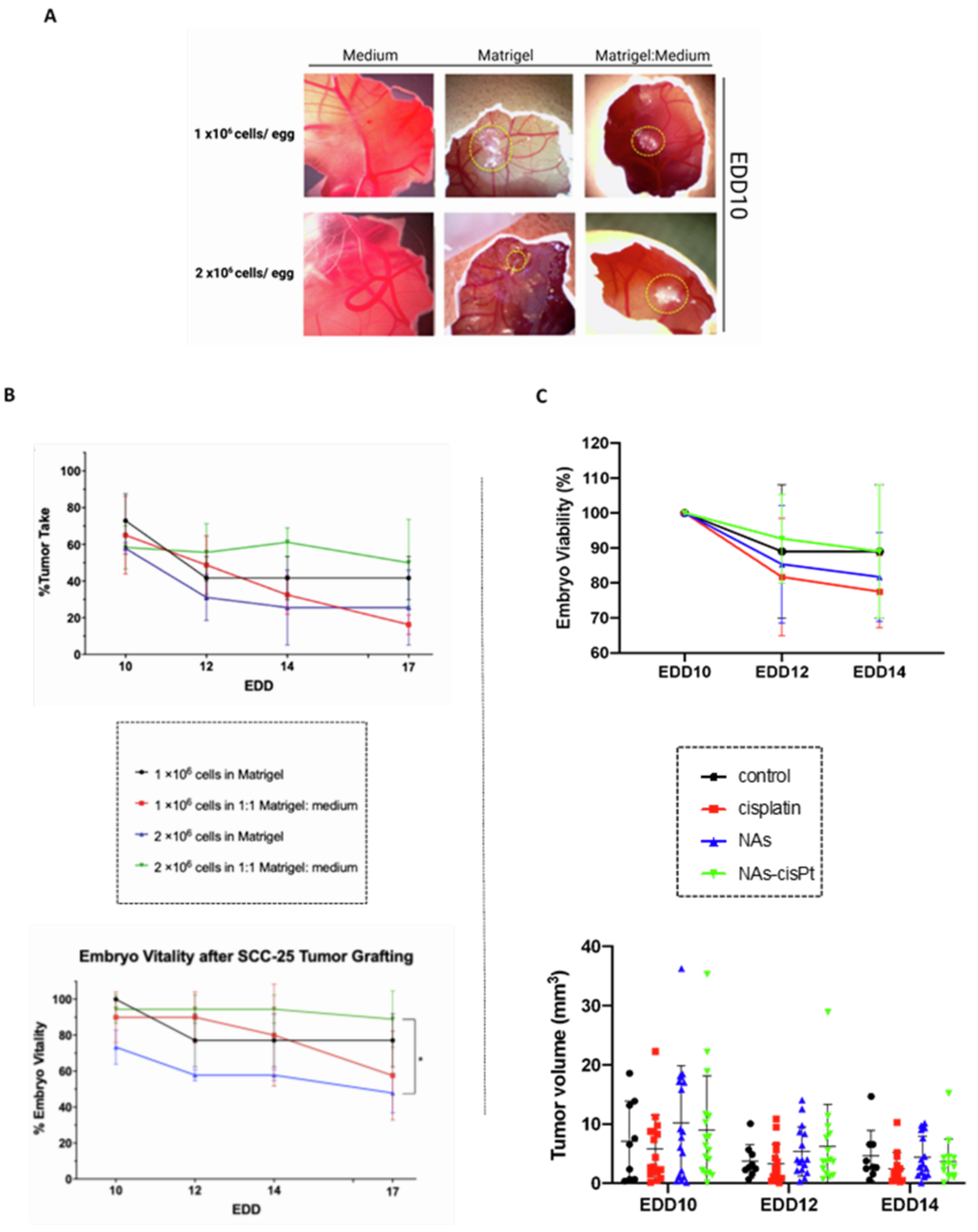

Figure S1. A) Representative images of tumors (yellow dashed circles) taken at EDD10 (4 days post-grafting). The deposition of 1×10^{6} or 2×10^{6} cells/egg with medium alone did not lead to tumor development. *B, top)* Tumor take rate after different grafting approaches. The tumor take rate was calculated by comparing the number of eggs in which tumor masses have been observed and the number of eggs

originally grafted with SCC-25 cells at EDD6. No statistical differences among the conditions were identified after data analysis. *B, bottom)* Viability of the embryo after grafting of SCC-25 cells. Significant difference was only observed at EDD 17 between 2×10^6 cells in Matrigel and 1:1 medium:Matrigel. Two-way ANOVA (Tukey's multiple comparison's test), $^*p < 0.05$ (*Data are reported as mean ± standard deviation of 2 independent experiments, with at least 4 eggs per condition, per experiment*). *C, upper)* Embryos vitality before (EDD10) and after treatment (EDD12-14). Data are reported as mean ± standard deviation of 3 independent experiments. *C, bottom)* Tumor volume before (EDD10) and after treatment (EDD12-14). Each point represents a single tumor mass. The middle bars represent the mean, while the top and bottom bars represent the standard deviation. Data are reported as mean ± standard deviation of 3 independent experiments.

Figure S2. Harvested tumors at EDD14, related to Figure 1.

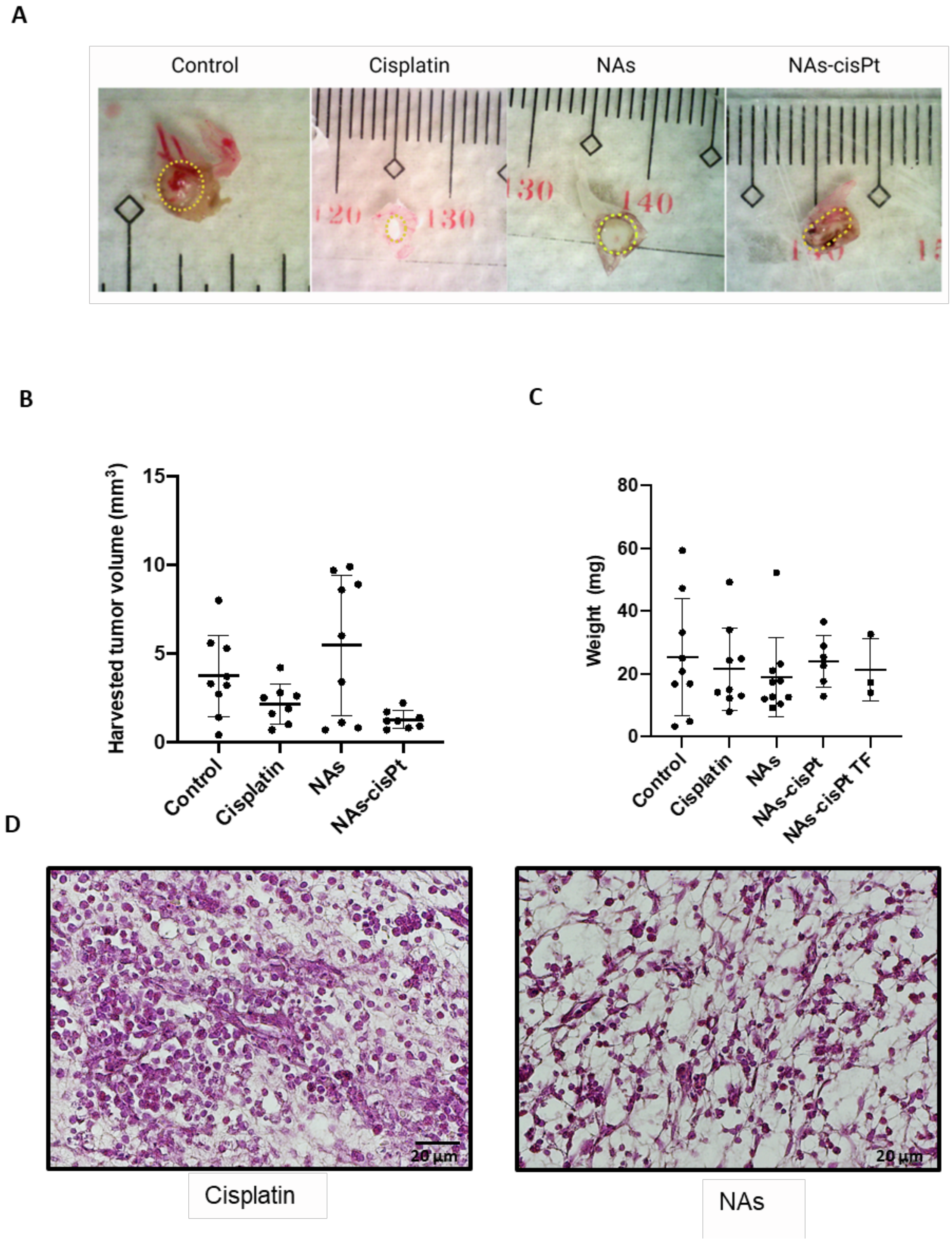

Figure S2. A) Tumors (yellow dashed circles) were collected at EDD14 and photographed to compare the sizes with their respective superficial measurements. *B)* The images were processed to take tumor dimensions and calculate the volumes*. C)* The weights of the collected tumors depend on the accuracy of the resection technique. After harvesting, the tumors were stored or processed for further experiments. Middle bars represent the mean of the measurements, while the top and bottom bars correspond to the

standard deviation values. *D)* Hematoxylin and Eosin imaging reveal the presence of undamaged cells in other areas of the tissues treated with cisplatin and NAs (Magnification 40X; scalebar 20 μm).

Table S1. Amounts (ng) of gold and platinum quantified in harvested organs, related to Figure 5.

A. Gold

	Liver	Heart
Control	2.39 ± 2.52	39.12 ± 0.62
Cisplatin	3.89 ± 2.33	38.66 ± 1.74
NAs	1.37 ± 0.21<br>(%AD = 0.006 ± 0.001)	46.90 ± 12.38<br>(%AD = 0.20 ± 0.05)
NAs-cisPt	5.08 ± 6.08<br>(%AD = 0.023 ± 0.028)	39.57 ± 1.20<br>(%AD = 0.16 ± 0.00_5)

B. Platinum

	Liver	Heart
Control	0.47 ± 0.39	6.80 ± 0.05
Cisplatin	9.01 ± 6.64<br>(%AD = 0.22 ± 0.17)	8.68 ± 1.42<br>(%AD = 0.22 ± 0.04)
NAs	0.11 ± 0.08	6.70 ± 0.01
NAs-cisPt	2.01 ± 1.22<br>(%AD = 0.05 ± 0.03)	6.91 ± 0.04<br>(%AD = 0.17 ± 0.00_1)

*Data are reported as mean ± standard deviation (N at least 2).
